# Watch the target! Effects in the affective misattribution procedure become weaker (but not eliminated) when participants are motivated to provide accurate responses to the target

**DOI:** 10.3389/fpsyg.2015.01442

**Published:** 2015-09-24

**Authors:** Andreas B. Eder, Roland Deutsch

**Affiliations:** ^1^Department of Psychology, University of WürzburgWürzburg, Germany; ^2^Fachrichtung Psychologie, Fakultät für Mathematik und Naturwissenschaften, Technische Universität DresdenDresden, Germany

**Keywords:** affect misattribution procedure, accuracy motivation, response priming, implicit attitude measurement

## Abstract

Previous research showed that priming effects in the affective misattribution procedure (AMP) are unaffected by direct warnings to avoid an influence of the primes. The present research examined whether a priming influence is diminished by task procedures that encourage accurate judgments of the targets. Participants were motivated to categorize the affective meaning of nonsense targets accurately by being made to believe that a true word was presented in each trial and by providing feedback on (allegedly) incorrect responses. This condition produced robust priming effects. Priming was however reduced and less reliable relative to more typical AMP conditions in which participants guessed the meaning of openly presented nonsense targets. Affective judgments of nonsense targets were not affected by advance knowledge of the response mapping during the priming phase, which argues against a response-priming explanation of AMP effects. These findings show that affective primes influence evaluative judgments even in conditions in which the motivation to provide accurate responses is high and a priming of motor responses is not possible. Priming effects were however weaker with high accuracy motivation, suggesting that a focus on accurate judgments is an effective strategy to control for an unwanted priming influence in the AMP.

## Introduction

During the last three decades, indirect measures of attitudes, stereotypes, and the self, have elicited immense interest in social psychology (for reviews see Petty et al., 2009; Gawronski and Payne, 2010; Roefs et al., 2011). A defining feature of indirect measurement procedures is that they imply variability in mental constructs (e.g., attitudes, stereotypes, self) from variability in judgments that are not about the mental construct under investigation (De Houwer et al., 2009). An indirect attitude measure that has become increasingly popular during the last few years is the affect misattribution procedure (*AMP*; Payne et al., 2005; for a review see Payne and Lundberg, 2014). In the AMP, participants watch a series of prime images or words, and each prime is followed by an ambiguous target (such as a Chinese character) in rapid succession. Participants are asked to rate the target in terms of its esthetic pleasantness or unpleasantness while they should ignore the primes. Despite these task instructions, target ratings are typically biased by the pleasantness of the primes: Participants judge targets that are presented after a pleasant prime as more pleasant than targets that appeared after unpleasant stimuli. This biasing influence was explained with an automatic evaluative response to the prime that is unintentionally misattributed to the target (Payne et al., 2010).

### Affect misattribution in the AMP: unavoidable or not?

Payne et al. (2005) conceptualize misattribution as “mistaking an effect of one source for the effect of another” (p. 278). They argue that people have difficulty disentangling their affective responses to two events occurring in close proximity in time and space. In line with this theorizing, it was shown that the magnitude of priming increases when the time interval between prime and target decreases and when the target is visible for only a short duration, presumably because participants have less time in these conditions to sort out reactions to the primes from reactions to the target. Furthermore, AMP-effects are stronger when participants are explicitly instructed to rely on their intuitive feelings during the judgment task (De Houwer and Tucker Smith, 2013). More important for the present discussion, target judgments are biased by the primes even after direct warnings of an influence of the primes (Payne et al., 2005). Latter finding suggests that participants are unable to correct for the influence of the primes even if they are instructed to do so (for a review see Payne and Lundberg, 2014).

Research on social judgments, on the other hand, has also pointed out that most judgmental biases reflect not so much a lack of ability but rather a motivation to use a biased set of cognitive strategies to solve a judgmental problem (Kruglanski, 1989). Accuracy-motivated people typically expend more cognitive effort on problem-related reasoning, attend to relevant information more carefully, and process it more deeply, often using more complex decision rules (Kunda, 1990). These strategies were effective in reducing judgmental biases in many tasks. For instance, experiments showed that social judgments of an ambiguously described target person are less consistent with primed constructs when participants had a strong goal to reach an accurate impression about the target relative to a standard condition in which participants were not explicitly instructed to be accurate (e.g., Thompson et al., 1994; Ford and Kruglanski, 1995). Judgmental biases produced by priming can thus be counteracted in priming tasks when people are motivated to use cognitive strategies that are more appropriate to deal with an unwanted priming influence. Blatant warnings against potential biases and direct instructions to correct for them were most often ineffective in this respect because they are too subtle for a sustained accuracy motivation or they lead people to rely even more on faulty judgment strategies (Wegner, 1994; Wegener and Petty, 1997). Task procedures that motivate accurate responses to the target on a sustained level may hence be more effective to reduce a priming bias in the AMP, provided that participants have appropriate control strategies at their disposal that can be accessed at will (see Wilson and Brekke, 1994, for a general discussion of this point).

The present research examined this hypothesis by increasing the participants' motivation to provide accurate judgments of the targets. The typical AMP emphasizes intuitive judgments and no error feedback is given because judgments based on personal taste are required. These task features encourage spontaneous judgments on the basis of momentary feeling because there is no penalty (e.g., error feedback) for confusion of the affective source. As a consequence, participants rely more on their intuitive feelings in order to come up with a meaningful response to a meaningless stimulus. The motivational setting is however different when responses to the target could be correct and incorrect. In such a task setting, an incorrect response to the target creates some cost (i.e., the experience of an error). Independent research suggests that participants are generally motivated to avoid making errors (Hajcak and Foti, 2008). To the degree that participants are motivated to report correct evaluations of the targets, they should hence use cognitive strategies that are better suited to disentangle affective a primes and targets—or fail in their correction attempts if no such strategies exist.

### Automatic response priming

Even if participants are motivated to provide accurate responses, it is possible that they are unable to do so due to an automatic priming of motor responses. Such response priming is viable in a standard AMP due to a fixed mapping of the evaluative response options onto the response keys: Throughout an experimental session, one response key is pressed if the target is rated as positive, and the other response key is pressed if the target is rated as negative. As a consequence, positive primes may activate the response associated with a positive judgment and negative primes may activate the response corresponding with a negative judgment, even before processing of the target (Gawronski et al., 2011; Eder et al., 2012). In support of this hypothesis, Scherer and Lambert (2009) observed that affectively neutral primes act like positive primes in the AMP when they are presented with clearly negative primes, whereas they act like positive primes when they are presented with clearly negative primes. The authors explained this contrast effect with a response-mapping framework: Participants automatically impose a good/bad classification scheme onto the primes, even when some of them are actually neutral. As a consequence, neutral primes elicit a positive key response when presented with negative primes and a negative key response when presented with positive primes.

A recent study, however, provided evidence against a response-priming explanation of AMP-effects. Gawronski and Ye (2014) presented a modified version of an AMP in which the assignment of the target responses to the response keys was randomly determined for each trial, preventing an advance preparation of key responses. Priming effects on evaluative and semantic target responses occurred regardless of whether the key assignment in the task was fixed or random. This (null) finding argues against a motor priming as the main cause of AMP-effects. A priming of motor responses, however, is still plausible for priming tasks in which participants prepare for categorizations of affectively polarized targets with a fixed response mapping (Eder et al., 2012). Therefore, we manipulated the response mapping in a related way to control for a contribution of response priming to AMP effects in our conditions.

### The present research

To manipulate the motivation to provide accurate responses to the targets, we had participants work on a modified AMP in *evaluation conditions* vs. *word-identification conditions*. In the evaluation conditions, native German speakers were instructed to make spontaneous evaluative guesses about the meaning of (allegedly Turkish) nonsense words whilst ignoring the presentation of affective primes (angry and happy faces). Thus, participants were explicitly informed about the presentation of targets they could not decode semantically, similar to the Chinese characters typically used in the AMP. And like in a standard AMP, it was emphasized that they should follow their spontaneous affective impressions during the judgment task. Importantly, no error feedback was provided in this condition. This condition hence resembles a standard AMP in which judgments about stimuli could be neither correct nor incorrect. In addition to a *standard-evaluation condition* with nonsense targets only, we also realized a *mixed-evaluation condition* in which participants categorized the valence of randomly intermixed positive and negative words in addition to evaluations of nonsense targets. This condition was implemented to direct the participants' attention to the targets, but here without explicit error feedback and instructions to provide accurate judgments.

In the *word-identification conditions*, by contrast, task instructions stated that a familiar German word is presented in every trial, when in fact only a few trials involved the presentation of meaningful German words. More specifically, German words with a clear positive or negative valence were presented in a subset of the block trials for an individually adjusted time that allowed for a correct (but imperfect) identification above chance. In the majority of the trials, however, meaningless letter strings were presented as targets for a very brief time that did not allow for a conscious reading of the word. Trials with presentations of meaningless letter strings were intermixed in a random and unpredictable order, so that participants had to stay focused on the targets in all trials for optimal performance. Participants were instructed to categorize the evaluative meaning of the target word as correctly as possible and to guess the target valence if they could not read the target. In addition, participants received an error feedback if the valence judgment was (allegedly) correct or incorrect (i.e., even after presentations of nonsense targets). We hypothesized that participants are motivated to categorize the valence of targets (and not the primes) when they receive feedback about correct and incorrect responses and when they believe that a known word is presented in each trials. Accordingly, participants should attempt to reduce intrusions by the prime, selecting cognitive strategies that are less affected by their presentations.

The role of response priming was examined by manipulating advance knowledge of the response-mapping rules in a similar way as Gawronski and Ye (2014) did in their study: In a *fixed-identification condition*, the mapping of the affective judgments onto the response keys was constant. One response key always indicated a positive word, while the other response key indexed a negative word judgment. In a *variable-identification condition*, however, the assignment of the affective judgment to the response keys could change from trial to trial, and participants did not know in advance which key they must press in order to indicate a positive or negative target. Given that direct activation of the classification response through the prime requires advance knowledge of the response mapping response priming should be decreased in the variable-identification condition relative to the fixed-identification condition. If response priming is not a main cause of AMP-effects, no difference between these conditions should be observed.

To summarize, following hypotheses were examined:

(1) Participants should rely less on their intuitive feelings evoked by the primes when they are penalized for incorrect responses and when they attempt to provide accurate responses to the targets. Priming effects should hence be reduced in the *word-identification conditions* relative to the *evaluation conditions* that encourage a more lenient response criterion. However, a priming effect should still be obtained in the *word-identification conditions* if affective feelings elicited by the primes are used automatically (i.e., unintentionally) for target judgment.
(2) If response priming is a main cause of AMP-effects, the size of priming should be increased in the *fixed-identification condition* in which participants have advance knowledge of the response mapping relative to the *variable-identification condition* in which participants do not know the assignment of the categorization response to the response keys before the priming phase. No difference between both conditions is expected if response priming does not substantially contribute to the AMP-effect, as concluded by Gawronski and Ye (2014).

## Methods

### Participants

A total of 160 students (94 women, *M* age = 23.7 years, *SD* = 5.3) with different majors participated in fulfillment of course requirement or for payment. All participants had normal or corrected-to-normal vision and were fluent in German. None was proficient in Turkish language. Forty participants were assigned to each of four different experimental conditions: (1) a condition with a fixed response mapping in which they categorized the valence of familiar German words and guessed the valence of nonsense (allegedly German) words that were presented too briefly for conscious identification (*fixed-identification condition*); (3) an analogous word-identification condition in which word categorizations were carried out with a variable response mapping (*variable-identification condition*); (3) a standard AMP condition in which they guessed the valence of nonsense (allegedly Turkish) words with a fixed response-mapping (*standard-evaluation condition*); (4) an analogous evaluation condition in which they categorized additionally the valence of intermixed familiar German words (*mixed-evaluation condition*). Participants signed a written informed consent before participation. Previous studies using similar procedures with affectively polarized primes obtained strong AMP-effects with standardized effect sizes (Cohen's *d*) ranging from 1.38 to 2.44 (Payne et al., 2005). Therefore, we collected data from 40 participants for each condition, which provides sufficient power to detect a strong AMP effect (*P* = 0.97).

### Design

The experimental design in the word-identification and in the mixed-evaluation conditions was a 2 (prime: happy vs. angry) × 3 (target: positive vs. negative vs. nonsense) factorial design. Each participant worked through 8 experimental blocks. Each block consisted of 16 trials with nonsense targets (preceded by 8 happy and 8 angry primes), 4 trials with positive targets and 4 trials with negative targets (preceded by 2 happy and 2 angry primes, respectively), resulting in 24 trials per block that were intermixed in random order. Nonsense targets (drawn from the practice set) replaced positive and negative target words in the standard-evaluation condition (i.e., only nonsense targets were presented in this condition), resulting in a 2 (prime: happy vs. angry) × 1 (target: nonsense) factorial design.

### Apparatus and stimuli

Prime stimuli were 48 happy faces and 48 angry faces that were selected from a standardized picture set (KDEF; Lundqvist et al., 1998). Half of the displayed faces were female, the other half showed male persons. Affective target stimuli were 32 clearly positive (*M* = 2.2, *SD* = 0.37) and 32 clearly negative adjectives (*M* = −2.2, *SD* = 0.48) that were selected from a standardized word pool on the basis of their evaluative norms (Schwibbe et al., 1981). The subsets of positive and negative adjectives did not differ in valence extremity, frequency of usage, or number of letters (range: 5–9), with all *Fs* < 1. Neutral target stimuli were 64 nonsense words (e.g., muganni) of ascending length (range: 5–9 letters). An additional 12 nonsense words were created for task practice. Response keys were the two buttons of a computer mouse.

### Procedure

#### Word-identification conditions

Participants completed first an adjustment phase and then a test phase. In the adjustment phase, the presentation duration of meaningful target words was individually adjusted to establish an ambiguous word identification task for the test phase. Participants performed five blocks with 12 trials each that involved the presentation of a positive or negative adjective with equal probability (i.e., only familiar words with a clear positive or negative valence were shown in this phase). Target words were the same adjectives that were later presented as targets in the experimental phase. They were randomly drawn from the word pool without replacement. Each trial started with the brief presentation (100 ms) of an asterisk as a fixation mark in the middle of the screen. After an additional interval of 100 ms, a white premask (nine X) was presented for one screen refresh cycle (14 ms), immediately followed by the adjective that stayed on the screen for an individually set presentation time (starting with 114 ms in the first block). The target word was followed by a white postmask (nine X) for 100 ms, followed by a blank screen for 250 ms. A judgment screen then appeared that asked the participant for his or her valence judgment with a corresponding left or right mouse button press depending on the experimental condition (see details on the response-mapping below). An arbitrary time limit of 2 s was set for the judgment but no emphasis was put on the speed of the response. At the end of a trial, participants were informed about incorrect valence judgments and/or time limit violations if any. The next trial started after 800 ms.

After each block, the word presentation time was adjusted using a staircase procedure to achieve a valence identification rate ranging between 7 and 10 correct identifications in a block (for a similar procedure see Eder and Klauer, 2007). The presentation time was decreased by one screen refresh cycle (14 ms) when the error rate was equal or lower than 16%. It was increased by one cycle when the error rate was equal or above 41%. The final presentation time was computed by averaging across presentation times of the last three blocks (rounded up or down to the next multiple of the refresh cycle).

**Figure 1:**
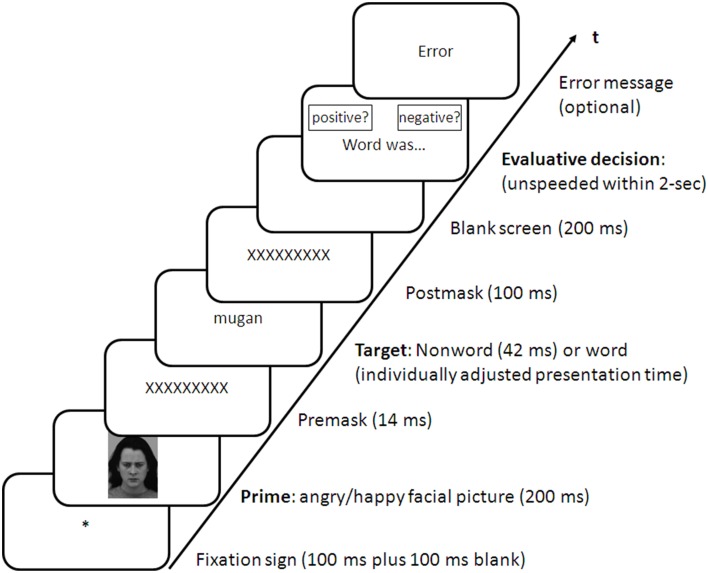
**Sequence of events in an experimental trial in the word-identification conditions**. Evaluative decisions were entered with left and right mouse button presses. The meaning of a key press was indexed by the respective location of response labels on left and right positions at the computer screen. In the fixed word-identification condition, the location of the response labels (i.e., the response mapping) was constant. In the variable word-identification condition, the location of the response labels varied unpredictably from trial to trial (see text for further explanation).

The sequence of events in the subsequent test phase was identical with that of the identification task in the adjustment phase, with the exceptions that (1) a picture of a facial emotional expression was now presented before the word and (2) the presentation of nonsense target stimuli in the majority of trials that were presented too briefly for conscious identification. Figure 1 shows the sequence of events in this phase. Participants were informed in the task instructions that a picture will now appear before the word. However, it was also stated that the picture is completely irrelevant for that task at hand (i.e., word categorization), and that it should therefore be ignored. The final word presentation time of the adjustment phase set the presentation duration of the positive and negative words in the first experimental block but was still adjusted (if necessary) after each block according to the staircase procedure detailed above. The presentation time of the nonsense targets was fixed to brief 42 ms, which was too brief for a conscious identification of the sandwich-masked stimuli (see Eder and Klauer, 2007). An incorrect valence judgment was reported back in half of these trials to maintain the illusion of a meaningful word presentation in every trial. Error feedback was still veridical in the trials with presentations of words as targets.

Judgmental responses to the target stimuli were entered differently in the experimental conditions. In the *fixed-mapping condition*, one mouse button was pressed to indicate a positive target meaning, while the other mouse button indicated a negative target meaning (counterbalanced assignment) throughout the experimental session, which corresponds with a typical affect-misattribution procedure. In the *variable-mapping condition*, the same mouse buttons were used for the target judgment but the assignment of the classification responses could vary from trial to trial. Participants were informed about the response mapping at the time of the judgmental response and each of the two assignments appeared with equal probability in each block (for details see Eder and Klauer, 2007). Thus, participants could not anticipate the motor response that corresponds with the prime valence during the priming phase.

Following the test phase, participants were probed for their awareness of the nonsense targets in the word-identification conditions with following questions: (i) “Which hypotheses do you think were investigated in this study?” (ii) “Did you have a strategy for the evaluation of the target?” (iii) “Did you recognize any words that were presented near to the perceptual threshold? If yes, what words?” (iv) “Did you encounter any problems?” A final question asked directly whether an adjective appeared in every trial, which was answered by selecting “yes” or “no.”

#### Evaluation conditions

No adjustment phase was necessary for these conditions. Instead, the test phase started immediately following six practice trials that illustrated the judgment procedure with clearly visible nonsense stimuli. For the *standard-evaluation condition*, the presentation time of the targets was fixed to brief 200 ms (without adjustments) and feedback was provided only on late responses. In the *mixed-evaluation condition*, 4 trials with positive and 4 trials with negative target words were intermixed in a trial block (see *Design* above) and no feedback was provided on the correctness of the valence categorization in these trials. The mapping of the valence categorization to the mouse buttons was fixed in both evaluation conditions (counterbalanced across participants).

## Results

Priming effects were calculated by subtracting the proportion of positive judgments on trials with a negative prime from the proportion of positive responses on trials with a positive prime. Internal consistency of the priming scores was assessed for each condition with the use of an odd-even split that divided the trials with positive and negative primes in two subsets depending on whether these trials were associated with an odd or even trial number (cf. Gawronski et al., 2010). For all analyses, the significance criterion was set to *p* < 0.05 (two-tailed). Standardized effect size (Cohen's *d*) and the 0.95 confidence intervals of the effect size are reported when appropriate. Analyses of the post-experimental suspicion measure are reported in an Appendix.

### Valence categorization of nonsense targets

**Figure 2:**
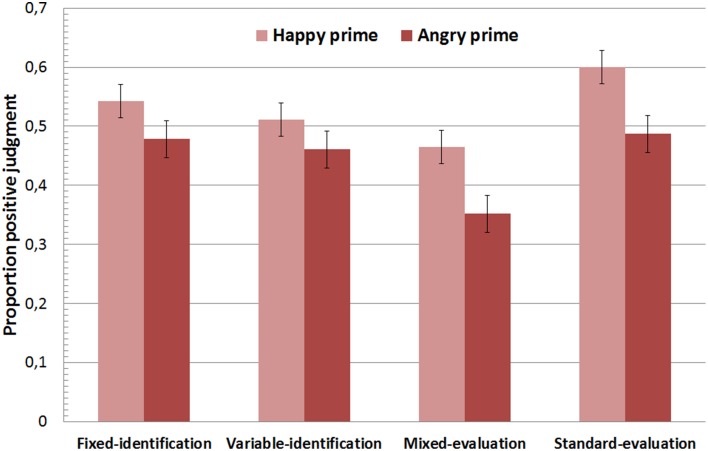
**Proportion of positive categorizations of nonsense targets as a function of affective prime and experimental condition**. Error bars display the 95% CI of the mean value.

The proportion of positive judgments in trials with nonsense targets was analyzed using a mixed 2 (prime) × 4 (condition) analysis of variance (ANOVA). The main effect of condition was significant, *F*_(1, 156)_ = 5.44, *p* < 0.05, *d* = 0.65. The proportion of positive judgments was lower in the mixed-evaluation condition relative to the remaining conditions. More important, the main effect of prime reached significance. As displayed in Figure 2, participants were more likely to judge a nonsense word as positive following a happy face compared to an angry face in every condition, *F*_(1, 156)_ = 53.61, *p* < 0.001, *d* = 1.17. The interaction between both factors missed significance, *F*_(1, 156)_ = 1.92, *p* = 0.13.

Follow-up tests of AMP-effects (difference between prime-congruent and prime-incongruent judgmental responses) against zero were significant in the *fixed-identification condition* (Δ*M* = 0.06, *d* = 0.74, 95% CI [0.39, 1.09]), *t*_(39)_ = 4.67, *p* < 0.001; in the *variable-identification condition* (Δ*M* = 0.05, *d* = 0.49, 95% CI [0.16, 0.82]), *t*_(39)_ = 3.09, *p* < 0.01; in the *mixed-evaluation condition* (Δ*M* = 0.12, *d* = 0.50, 95% CI [0.16, 0.83]); and in the *standard-evaluation condition* (Δ*M* = 0.11, *d* = 0.90, 95% CI [0.53, 1.26]), *t*_(39)_ = 5.72, *p* < 0.001. Thus, affective priming was observed in all conditions including the word-identification conditions (Hypothesis 1). Reliability of the priming scores was however insufficient in the *fixed-identification* and *variable-identification conditions* with *r* = 0.04 and *r* = 0.15, respectively; in contrast, priming effects were reliable in the *mixed-evaluation condition: r* = 0.75 (*p* < 0.001), and in the *standard-evaluation condition: r* = 0.66 (*p* < 0.001).

An additional analysis was performed to examine an influence of accuracy motivation on the size of priming effects. Experimental conditions were dummy-coded into those with a high motivation to provide accurate responses (the word-identification conditions) and those with a low motivation to respond accurately (the evaluation conditions). A *t*-test (independent samples) revealed stronger AMP effects in the evaluation conditions relative to the word-identification conditions, *t*_(158)_ = 2.38, *p* < 0.05, *d* = 0.38. In short, effect size was reduced in conditions with a high motivation to provide accurate responses (Hypothesis 1). Priming effects in the *standard-evaluation condition* and in the *mixed-evaluation conditions* were not different (*t* < 1). A comparison of effects in the *fixed-identification* and the *variable-identification conditions* also produced no difference (*t* < 1). Thus, effect sizes were not different with advance knowledge of the response mapping, suggesting that automatic response priming does not contribute to affective priming in the AMP (Hypothesis 2).

### Valence categorization of words

Rates of correct word categorizations in the word-identification conditions and in the mixed-evaluation condition were analyzed for a priming influence and whether participants in the word-identification conditions followed instructions to categorize the targets. The mean adjusted presentation duration for the affective words in the word-identification conditions was 75 ms (*SD* = 28 ms). The mean proportions of correct word evaluations were 74% (*SD* = 7.1) in the *variable-identification condition* and 72% (*SD* = 6.3) in the *fixed-identification condition* without significant difference, *t*_(78)_ = 1.04, *p*>0.10. The identification rates were however significantly higher than chance (*P* = 50%) in both conditions, *t*_(39)_ = 21.36, *p* < 0.001, *d* = 3.38, and *t*_(39)_ = 22.53, *p* < 0.001, *d* = 3.56, confirming that participants categorized targets. Accuracy in the *mixed-evaluation condition* with a fixed presentation time of the affective word was at a comparable level (*M* = 76%, *SD* = 14.7), *t*_(39)_ = 11.01, *p* < 0.001, *d* = 3.53.

A priming influence on affective word categorizations was analyzed using a 2 (prime valence) × 2 (target valence) × 3 (condition: fixed identification vs. variable condition vs. mixed-evaluation) ANOVA. Table 1 shows the proportion of correct target categorizations in each priming condition. The main effect of target valence was significant, indicating more accurate categorizations of positive words, *F*_(1, 117)_ = 4.30, *p* < 0.05, *d* = 0.38. The prime × target interaction, *F*_(1, 117)_ = 10.58, *p* < 0.01, *d* = 0.60, and the three-way interaction reached also significance, *F*_(1, 117)_ = 3.65, *p* < 0.05, *d* = 0.50. All other effects were not significant (with *p*s > 0.20). Follow-up tests of priming effects in each condition revealed significant priming effects in the *mixed-evaluation condition, F*_(1, 39)_ = 8.02, *p* < 0.01, *d* = 0.91, and in the *fixed-identification condition, F*_(1, 39)_ = 6.06, *p* < 0.05, *d* = 0.79, but not in the *variable-identification condition* (*F* < 1). Thus, affective priming of word categorizations was observed only in the conditions with a fixed-response mapping.

**Table 1:** **Proportion of correct evaluations of positive and negative target words (in percent) as a function of prime valence and experimental condition**.

	**Variable-identification**		**Fixed-identification**		**Mixed-evaluation**	
	**Positive target**	**Negative target**	**Positive target**	**Negative target**	**Positive target**	**Negative target**
Happy prime	74.2 (15.5)	74.5 (14.3)	78.0 (9.9)	68.6 (12.7)	83.1 (20.7)	67.5 (24.0)
Angry prime	73.0 (15.9)	73.9 (12.4)	72.3 (13.3)	70.5 (16.3)	74.4 (25.9)	77.3 (15.0)

*Standard deviation in parentheses*.

## Discussion

The present research examined whether participants could escape from priming in a modified AMP task when they have a motivation to provide accurate responses to the targets. In the word-identification conditions, participants were motivated to categorize targets by being made to believe that a familiar word was presented in each trial and by providing error feedback on (allegedly) inaccurate categorizations. Results showed that evaluative categorizations of masked nonsense targets were affected by prior presentations of task-irrelevant happy and angry facial picture primes: Meaningless letter strings (presented too briefly for conscious identification) were judged more often positive when they appeared after happy primes relative to angry primes. Furthermore, participants categorized clearly visible positive and negative words correctly, showing that participants were indeed motivated to respond to the targets. In combination, these findings demonstrate that affective priming is observed even in conditions in which participants have a motivation to reduce a biasing influence by the primes, supporting previous conclusions that a misattribution of affective responses from the primes to the targets is unintended (Payne et al., 2005).

However, a comparison of different AMP conditions also showed that priming effects in the word-identification conditions were diminished and less reliable relative to typical AMP conditions in which participants were aware of presentations of nonsense targets (evaluation conditions). This difference shows that a motivation to provide accurate responses can attenuate the influence of affective primes to some extent, suggesting that participants have at least partial control over a priming influence. From the present research, it cannot be concluded what mechanisms were responsible for the attenuated priming effects in the word-identification conditions. One possibility is that participants gave less weight to their spontaneous feelings during the judgment task, which may have an attenuating effect that is opposite to the one when participants are explicitly encouraged to rely more on their spontaneous feelings (De Houwer and Tucker Smith, 2013). A strategic weighting of “gut feelings” may hence work in both directions. Alternatively, it is possible that the categorization task and the emphasis on accuracy induced an analytical thinking mode that is known to disrupt holistic impressions and intuitions based on affective cues (e.g., Wilson and Schooler, 1991; Halberstadt, 2010). Another possibility is that the error feedback triggered behavioral adjustments that minimize a priming influence in the next trial. For instance, Frings and Wentura (2008) observed that affective priming in a valence categorization task is attenuated after incongruent trials (with typically more incorrect responses) relative to congruent trials. Sequential modulations of this sort are typically explained with reactive behavioral adjustments following conflict and/or errors that reduce interference by irrelevant primes (Dignath et al., 2015). Reactive adjustments on a trial-to-trial basis can also explain why the internal consistency of the priming scores approached zero in the word-identification conditions. Strategic adjustments in cognitive control after error feedback can hence explain why the AMP-effect was attenuated and less reliable in the word-identification conditions. A third possibility is enhanced attention to the relevant target information, which is known to reduce priming effects in other priming tasks (e.g., Musch and Klauer, 2001). In respect to this explanation it should be noted, however, that priming effects were not attenuated in a mixed-evaluation condition that analogously directed the participants' attention to the targets (as indexed by the accurate judgments of intermixed affective words). Thus, an explanation with differences in selective attention only is not supported. More research is needed on the specific mechanisms underlying the impact of accuracy motivation on priming effects in the AMP.

Advance knowledge of the response mapping in the word-identification conditions had no effect on the size of the AMP effect, which replicates the result of an earlier study that concluded first that response-priming provides no viable explanation of priming effects in the AMP (Gawronski and Ye, 2014). Given the interpretation of a null finding, it is reassuring to know that the present study reaches the same conclusion with different material and with an increased sample size (the present study: *N* = 80; Gawronski and Ye: *N* = 55). However, analyses of correct word categorizations also showed significant affective priming effects only for the conditions with a fixed-response mapping and not for the condition with a variable-response mapping. It is unclear why advance knowledge of the response mapping influenced affective priming only in trials with affective word categorizations and not in trials with presentations of nonsense targets. Due to the random intermixing procedure, participants could not anticipate whether a word is presented after the prime, ruling out differences in the strength of response preparation as a possible explanation. Clearly, more research is needed on this unexpected finding.

The observation of a priming effect in conditions with a high accuracy motivation is also important in respect to a recent discussion about the implicit nature of AMP effects. Bar-Anan and Nosek (2012) concluded from a series of large-scale internet studies that the psychometric qualities of the AMP are related to participants' self-reported intention to evaluate the prime (instead of the target). Participants who reported that they intentionally categorized the primes exhibited larger effect sizes, higher reliability, and stronger associations with other attitude measures relative to people who were not aware of and/or ignored the primes. This research questioned the status of the AMP as an implicit measurement procedure of attitudes. However, another study provided evidence that participants made up a response strategy after their behavior to justify a judgment bias (Payne et al., 2013). Furthermore, Gawronski and Ye (2015) showed a reliable priming of affective judgments even when participants were unaware of affective responses to the primes. Thus, evidence is accumulating that affective primes influence judgments in the AMP even when participants are unaware of and/or motivated to control for an influence of the primes, confirming that the AMP has a potential for an implicit measurement of attitudes.

## Funding

This research was supported by a grant of the German Research Foundation (DFG) to AE (ED 201/2-2).

### Conflict of interest statement

The authors declare that the research was conducted in the absence of any commercial or financial relationships that could be construed as a potential conflict of interest.

## Appendix

### Suspicion of nonsense target presentations in the word-identification conditions

No participant indicated suspicion of nonsense targets in the post-experimental questionnaire. However, 18 (out of 40) participants in the *variable-identification condition* and 19 (out of 40) participants in the *fixed-identification condition* did not believe that a familiar word was presented in every trial. These participants were classified as being suspicious of presentations of nonsense targets. In an ANOVA of the priming effects (i.e., the difference in positive judgments following positive and negative primes) with condition (variable-identification vs. fixed-identification condition) and suspicion as between-subjects factors, only the interaction between condition and suspicion reached significance, *F*_(1, 76)_ = 4.76, *p* < 0.05, *d* = 0.50. Follow-up comparisons (Fisher's LSD test) revealed significantly stronger effects in the *variable-identification condition* when participants had no suspicion in respect to presentations of nonsense targets relative to suspicious participants (*p* = 0.02), while suspicion did not influence AMP-effects in the *fixed-identification condition* (*p* = 0.48). If anything, suspicion of nonsense targets in the word-identification conditions thus appeared to decrease the priming influence.

## References

[B1] Bar-Anan Y. Nosek B. A. (2012). Reporting intentional rating of the primes predicts priming effects in the affective misattribution procedure. Pers. Soc. Psychol. Bull. 38, 1194–1208. 10.1177/0146167212446835 22611055

[B2] De Houwer J. Teige-Mocigemba S. Spruyt A. Moors A. (2009). Implicit measures: a normative analysis and review. Psychol. Bull. 135, 347–368. 10.1037/a0014211 19379018

[B3] De Houwer J. Tucker Smith C. (2013). Go with your gut! Effects in the affect misattribution procedure become stronger when participants are encouraged to rely on their gut feelings. Soc. Psychol. 44, 299–302. 10.1027/1864-9335/a000115

[B4] Dignath D. Kiesel A. Eder A. B. (2015). Flexible conflict management: conflict avoidance and conflict adjustment in reactive cognitive control. J. Exp. Psychol. Learn. Mem. Cogn. 41, 975–988. 10.1037/xlm0000089 25528083

[B5] Eder A. B. Klauer K. C. (2007). Common valence coding in action and evaluation: affective blindness towards response-compatible stimuli. Cogn. Emot. 21, 1297–1322. 10.1080/02699930701438277

[B6] Eder A. B. Leuthold H. Rothermund K. Schweinberger S. R. (2012). Automatic response activation in sequential affective priming: an ERP study. Soc. Cogn. Affect. Neurosci. 7, 436–445. 10.1093/scan/nsr033 21642351 PMC3324576

[B7] Ford T. E. Kruglanski A. W. (1995). Effects of epistemic motivations on the use of accessible constructs in social judgment. Pers. Soc. Psychol. Bull. 21, 950–962. 10.1177/0146167295219009

[B8] Frings C. Wentura D. (2008). Trial-by-trial effects in the affective priming paradigm. Acta Psychol. 128, 318–323. 10.1016/j.actpsy.2008.03.004 18440483

[B9] Gawronski B. Cunningham W. A. LeBel E. P. Deutsch R. (2010). Attentional influences on affective priming: does categorisation influence spontaneous evaluations of multiply categorisable objects? Cogn. Emot. 24, 1008–1025. 10.1080/02699930903112712

[B10] Gawronski B. Deutsch R. Banse R. (2011). Response interference tasks as indirect measures of automatic associations, in Cognitive Methods in Social Psychology, eds Klauer K. C. Voss A. Stahl C. (New York, NY: Guilford Press), 78–123.

[B11] Gawronski B. Payne B. K. (eds.). (2010). Handbook of Implicit Social Cognition: Measurement, Theory, and Applications. New York, NY: GuilfordPress.

[B12] Gawronski B. Ye Y. (2014). What drives priming effects in the affect misattribution procedure? Pers. Soc. Psychol. Bull. 40, 3–15. 10.1177/0146167213502548 23982152

[B13] Gawronski B. Ye Y. (2015). Prevention of intention invention in the affect misattribution procedure. Soc. Psychol. Personal. Sci. 6, 101–108. 10.1177/1948550614543029

[B14] Hajcak G. Foti D. (2008). Errors are aversive: defensive motivation and the error-related negativity. Psychol. Sci. 19, 103–108. 10.1111/j.1467-9280.2008.02053.x 18271855

[B15] Halberstadt J. (2010). Intuition: dumb but lucky. Fortuitous affective cues and their disruption by analytic thought. Soc. Pers. Psychol. Compass 4, 64–76. 10.1111/j.1751-9004.2009.00242.x

[B16] Kruglanski A. W. (1989). The psychology of being “right”: the problem of accuracy in social perception and cognition. Psychol. Bull. 106, 395–409. 10.1037/0033-2909.106.3.395

[B17] Kunda Z. (1990). The case for motivated reasoning. Psychol. Bull. 108, 480–498. 10.1037/0033-2909.108.3.480 2270237

[B18] Lundqvist D. Flykt A. Öhman A. (1998). The Karolinska Directed Emotional Faces (KDEF). Stockholm: CD ROM from Department of Clinical Neuroscience, Psychology Section, Karolinska Institute.

[B19] Musch J. Klauer K. C. (2001). Locational uncertainty moderates affective congruency effects in the evaluative decision task. Cogn. Emot. 15, 167–188. 10.1080/02699930126132

[B20] Payne B. K. Brown-Iannuzzi J. Burkley M. Arbuckle N. L. Cooley E. Cameron C. D.. (2013). Intention invention and the affect misattribution procedure: reply to Bar-Anan and Nosek (2012). Pers. Soc. Psychol. Bull. 39, 375–386. 10.1177/0146167212475225 23401479

[B21] Payne B. K. Cheng C. M. Govorun O. Stewart B. D. (2005). An inkblot for attitudes: affect misattribution as implicit measurement. J. Pers. Soc. Psychol. 89, 277–293. 10.1037/0022-3514.89.3.277 16248714

[B22] Payne B. K. Hall D. L. Cameron C. D. Bishara A. J. (2010). A process model of affect misattribution. Pers. Soc. Psychol. Bull. 36, 1397–1408. 10.1177/0146167210383440 20837777

[B23] Payne K. Lundberg K. (2014). The affect misattribution procedure: ten years of evidence on reliability, validity, and mechanisms. Soc. Pers. Psychol. Compass 8, 672–686. 10.1111/spc3.12148

[B24] Petty R. E. Fazio R. H. Briñol P. (eds.). (2009). Attitudes: Insights From the New Implicit Measures. New York, NY: Psychology Press.

[B25] Roefs A. Huijding J. Smulders F. T. Y. MacLeod C. M. de Jong P. J. Wiers R. W.. (2011). Implicit measures of association in psychopathology research. Psychol. Bull. 137, 149–193. 10.1037/a0021729 21219060

[B26] Scherer L. D. Lambert A. J. (2009). Contrast effects in priming paradigms: implications for theory and research on implicit attitudes. J. Pers. Soc. Psychol. 97, 383–403. 10.1037/a0015844 19685997

[B27] Schwibbe M. Röder K. Schwibbe G. Borchardt M. Geiken-Pophanken G. (1981). Zum emotionalen gehalt von substantiven, adjektiven und verben [the emotional contents of nouns, adjectives, and verbs]. Z. Exp. Angew. Psychol. 28, 486–501.

[B28] Thompson E. P. Roman R. J. Moskowitz G. B. Chaiken S. Bargh J. A. (1994). Accuracy motivation attenuates covert priming: the systematic reprocessing of social information. J. Pers. Soc. Psychol. 66, 474–489. 10.1037/0022-3514.66.3.474

[B29] Wegener D. T. Petty R. E. (1997). The flexible correction model: the role of naive theories of bias in bias correction, in Advances in Experimental Social Psychology, Vol. 29, ed Zanna M. P. (New York, NY: Academic Press), 141–208.

[B30] Wegner D. M. (1994). Ironic processes of mental control. Psychol. Rev. 101, 34–52. 10.1037/0033-295X.101.1.34 8121959

[B31] Wilson T. D. Brekke N. (1994). Mental contamination and mental correction: unwanted influences on judgments and evaluations. Psychol.Bull. 116, 117–142. 10.1037/0033-2909.116.1.117 8078969

[B32] Wilson T. D. Schooler J. W. (1991). Thinking too much: introspection can reduce the quality of preferences and decisions. J. Pers. Soc. Psychol. 60, 181–192. 10.1037/0022-3514.60.2.181 2016668

